# Nonhomologous end joining—the importance of end tethering and beyond

**DOI:** 10.1101/gad.353407.125

**Published:** 2026-04-01

**Authors:** Shan Zha, Geunil Yi

**Affiliations:** 1 Institute for Cancer Genetics, Vagelos College for Physicians and Surgeons, Columbia University, New York, New York 10032, USA;; 2 Herbert Irvine Comprehensive Cancer Center, Vagelos College for Physicians and Surgeons, Columbia University, New York, New York 10032, USA;; 3 Department of Pediatrics, Vagelos College for Physicians and Surgeons, Columbia University, New York, New York 10032, USA;; 4 Department of Pathology and Cell Biology, Vagelos College for Physicians and Surgeons, Columbia University, New York, New York 10032, USA;; 5 Department of Microbiology and Immunology, Vagelos College for Physicians and Surgeons, Columbia University, New York, New York 10032, USA

**Keywords:** classical nonhomologous end joining, DNA double-strand breaks, DNA damage response, end processing, DNA repair, end tethering, NHEJ, V(D)J recombination

## Abstract

The nonhomologous end joining (NHEJ) pathway is a major mechanism for repairing DNA double-strand breaks (DSBs), essential for lymphocyte development and for maintaining genome stability in postmitotic cells, including neurons. Under pathological conditions, NHEJ is also responsible for most oncogenic translocations and the repair of DSBs induced by chemotherapy/radiation. Advances in structural biology and single-molecule imaging have captured NHEJ at various stages of the reaction, revealing a modular, dynamic organization with built-in redundancy and resilience. Here, we describe NHEJ in five phases (end sensing, end protection, end tethering, end processing, and end ligation), highlighting their molecular features and transitions and how structural insights explain genetic interactions and physiological consequences, including lymphocyte development. Unlike other DSB repair mechanisms, NHEJ operates with minimal or no sequence complementarity, relying instead on protein assemblies to bridge and stabilize the two DNA ends. This review highlights the unique end-tethering phase, supported by at least five known overlapping machineries, all anchored on Ku.

## Abstract (precis)

In this review, Zha and Yi dissect the molecular and structural mechanisms underlying the five discrete phases of nonhomologous end joining (NHEJ); namely, end sensing, end protection, end tethering, end processing, and end ligation. They highlight how the dynamic network of protein bridges (i.e., Ku–protein interactions) stabilizes DNA ends and enables the assembly of NHEJ machinery to facilitate the repair of DNA double-strand breaks independent of sequence complementarity.

DNA double-strand breaks (DSBs) represent the most severe form of DNA damage. Eukaryotic cells repair DSBs primarily through two pathways: homologous recombination (HR) and nonhomologous end joining (NHEJ). Among these, NHEJ directly ligates two DNA ends with minimal end processing and functions throughout the cell cycle, making it the major DSB repair mechanism in postmitotic somatic cells, including neurons (Lieber 2010; Ochi et al. 2014; Wang et al. 2020). Developing B and T cells rely on NHEJ exclusively to resolve DSBs generated during V(D)J recombination that occurs in the G0/G1 phase, and mature B cells also use NHEJ to complete immunoglobulin class switch recombination (CSR) (Wang et al. 2020). In the absence of NHEJ, ∼25%–50% of CSRs can proceed via an alternative end joining (A-EJ) pathway that preferentially uses microhomology (MH) at the junctions (also referred to as MH-mediated end joining [MMEJ]) (Corneo et al. 2007; Yan et al. 2007; Boboila et al. 2010; Wang et al. 2020). To distinguish it from A-EJ or MMEJ, the canonical pathway is sometimes referred to as classical NHEJ (C-NHEJ), which is the focus of this review. Several excellent reviews have discussed A-EJ/MMEJ and polymerase θ-mediated end joining, a subset of A-EJ (Ramsden et al. 2022; Sfeir et al. 2024). Given the aforementioned physiological roles of NHEJ, microcephaly, B^−^T^−^ severe combined immunodeficiency (B^−^T^−^-SCID), and growth retardation are frequently observed in patients and animal models with NHEJ deficiency (see below for details; Wang et al. 2020; Fournier et al. 2022). Beyond physiological repair, NHEJ also repairs DSBs from ionizing radiation and genotoxic chemotherapy and contributes to oncogenic chromosomal translocations (Ghezraoui et al. 2014).

The mammalian NHEJ pathway currently includes eight well-characterized members: the Ku70/Ku80 (Ku86 for humans) heterodimer (collectively referred to as Ku), X-ray repair cross-complementing protein 4 (XRCC4), ligase 4 (LIG4), XRCC4-like factor (XLF; also called Cernunnos; official name NHEJ1), paralog of XRCC4 and XLF (PAXX; also called C9ORF142), DNA-dependent protein kinase catalytic subunit (DNA-PKcs; official name PRKDC), and Artemis nuclease (official name DCLRE1C) (Lieber 2010; Ochi et al. 2015; Xing et al. 2015). In addition, three DNA polymerases (TdT, Polµ, and Polλ) directly interact with Ku and polish the ends during NHEJ. Finally, ERCC6L2 (Francica et al. 2020; Liu et al. 2020), CYREN (also called MRI) (Arnoult et al. 2017; Hung et al. 2018), APLF (Hammel et al. 2016; Nemoz et al. 2018), and WRN are also implicated in NHEJ through their interaction with Ku (Grundy et al. 2016; Nemoz et al. 2018), but their exact roles are yet to be determined. This review mostly focuses on the eight well-characterized members with detailed structural information and uses their conventional names, not always their official gene names, hereafter.

Among them, Ku70, Ku80, XRCC4, LIG4, XLF, and PAXX can be found from yeast to humans and are required for end ligation, while DNA-PKcs and Artemis are mostly found in vertebrates. While loss of DNA-PKcs or Artemis always abrogates end processing, and loss of Artemis in general does not abolish end ligation of blunt and clean ends, the impact of DNA-PKcs on end ligation varies (Blunt et al. 1995; Hartley et al. 1995; Gao et al. 1998; Taccioli et al. 1998). In mouse models, loss of DNA-PKcs abrogates end processing while having a limited impact on end ligation of blunt and clean DNA ends. The distinction is clearly illustrated in the context of lymphocyte development, where the RAG endonuclease initiates V(D)J recombination by generating two blunt signaling ends (SEs) and two hairpinned coding ends (CEs) (Alt et al. 2013). Loss of DNA-PKcs and Artemis abrogates hairpin opening—and thus the joining between two CEs to form the coding joints (CJs)—but does not abolish end ligation between the blunt SEs that form the signal joints (SJs) (Moshous et al. 2001; Rooney et al. 2002; Wang et al. 2020). Loss of Ku, XRCC4, LIG4, XLF, and PAXX alone or in combination compromises end ligation and therefore both CJ and SJ formation. Furthermore, some NHEJ factors, most notably Ku and DNA-PKcs, have functions beyond NHEJ, including telomere maintenance (Pfingsten et al. 2012; Dalby et al. 2013; Chen et al. 2018; Sonmez et al. 2024; Eickhoff et al. 2025), RNA processing (Dalby et al. 2013; Shao et al. 2020; Pascarella et al. 2025; Yu et al. 2025), and suppression of RNA-mediated innate immune response (Zhu et al. 2025), which are beyond the scope of this review.

NHEJ can be divided into five mostly discrete phases: end sensing, end protection, end tethering, end processing, and end ligation (Fig. 1A; Lieber 2010; Alt et al. 2013; Jette and Lees-Miller 2015). While end sensing and end protection initiate and end ligation concludes NHEJ, end processing occurs at discrete steps (see below), and end tethering functions throughout NHEJ. Unlike HR or A-EJ/MMEJ, NHEJ operates without sequence homology between the two DNA ends, instead relying on at least five known protein bridges (more is very possible; see the discussion at the end) to bring and hold the two ends together, referred to as end tethering in this review (Fig. 1B). While all of the five bridges are based on Ku, two involve DNA-PKcs, one involves PAXX, and two involve XLF. An important step within NHEJ is the release of DNA-PKcs from both ends, which denotes the transition from long-range to short-range NHEJ complexes observed in single-molecule and structural analyses (Ochi et al. 2014; Graham et al. 2016; Chaplin et al. 2020; Chen et al. 2021a,b; Liu et al. 2022). Among the two end-processing steps, Artemis-mediated endonuclease cleavage occurs in the long-range complex in the presence of DNA-PKcs on DNA ends. On the other hand, while the polymerase (µ, λ, and TdT) might be recruited via Ku earlier, the polymerase-mediated filling can only occur after DNA-PKcs displacement in the short-range complex with the help of DNA LIG4. For this reason, we divided end processing into parts I and II, flanking end tethering in between. This review highlights recent structural insights that provide molecular explanations for the genetic interactions within the NHEJ pathway members and their physiological implications.

**Figure 1.**
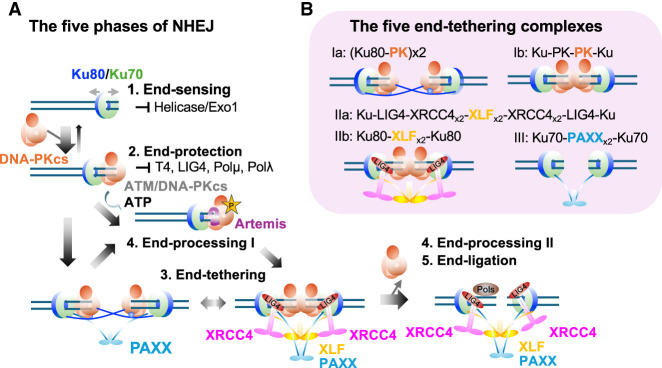
The five phases of NHEJ and the five molecular bridges. (*A*,*B*) The five phases of NHEJ. (*A*, part *1*) End sensing through Ku. (*A*, part *2*) End protection through Ku alone (prevents exonuclease resection and helicase separation) and via DNA-PKcs (caps the ends). DNA-PKcs binding pushes the Ku to rotate away from the ends. In the Apo form before kinase activation, DNA-PKcs protects the ends, preventing end ligation and end processing. (*A*, part *3*) End tethering via DNA-PKcs (*B*, panels *Ia*,*Ib*), via XLF (*B*, panels *IIa*,*IIb*), and via PAXX (*B*, panel *III*). (*B*, panels *IIa*,*IIb*,*III*) In the absence of DNA-PKcs, ends can be tethered via XLF and PAXX in a short-range complex compatible with end ligation. (*A*, part *4*) End processing. Phosphorylation of DNA-PKcs by itself or by ATM triggers additional conformation changes and further rotation inward to expose the ends and open up DNA-PKcs to bring in Artemis; in this form, DNA-PKcs recruits and activates Artemis for hairpin cleavage. DNA-PKcs needs to be activated (denoted by a star) to deprotect/present the ends. Then, the ends can be filled in, blunted, or have N nucleotides added. (*A*, part *5*) End ligation via XRCC4–LIG4. The XLF homodimer binds to the XRCC4 (also sometimes referred to as XR4 in the diagram) homodimer on each side and loads two LIG4s (one for each strand of DNA). In parallel, Polµ and Polλ can be recruited via direct interaction with Ku to fill in the ends. Among the five steps, end sensing, end protection, and Artemis-mediated end processing all occur in the context of the long-range complex with DNA-PKcs, while end ligation and polymerase fill-in occur in the short-range complex after DNA-PKcs dissociation. End tethering occurs throughout the long-range-to-short-range transition (see Fig. 2 for more details). (*B*) The five protein bridges that constitute the end-tethering complexes are as follows: via Ku–DNA-PKcs swap dimer and Ku–PK–PK–Ku bridge (panels *Ia*,*Ib*), via Ku80–XLF–Ku80 and Ku–LIG4–XRCC4–XLF–XRCC4–LIG4–Ku (panels *IIa*,*IIb*), and via Ku70–PAXX–Ku70 (panel *III*).

## End sensing

**Figure 2.**
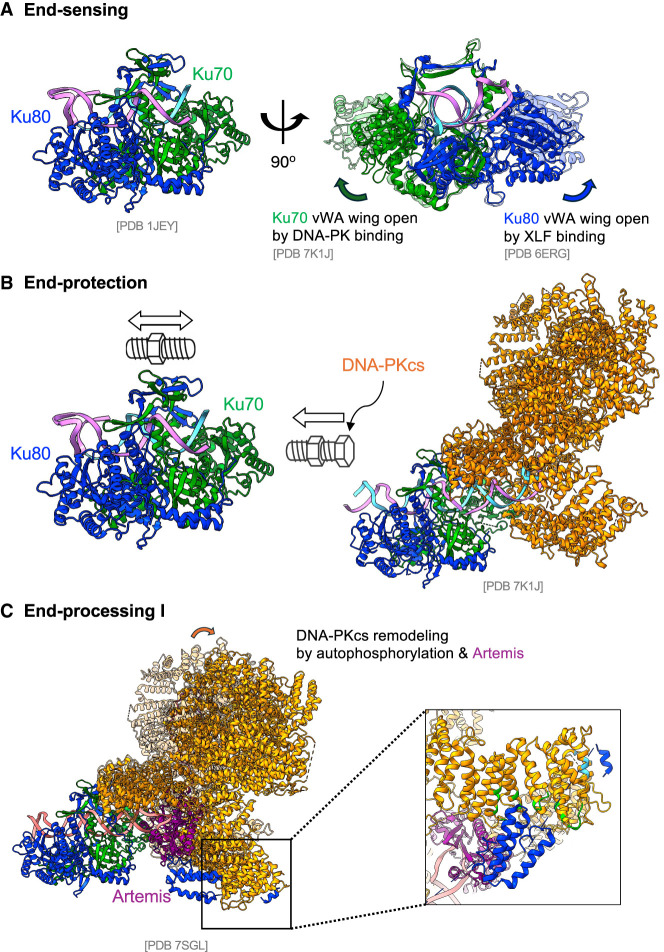
The molecular model for end sensing, end protection, and end processing I. (*A*) A modified structural model based on PDB 1JEY shows Ku binding to DNA ends, with Ku70 positioned proximal to the DNA terminus. The vWA domains of Ku70 and Ku80 form two “wings” that can adopt either uplifted or down positions. Binding of DNA-PKcs to Ku70 stabilizes the uplifted conformation of the Ku70 vWA domain and facilitates PAXX recruitment. Subsequently, the XLF tail binds beneath the Ku80 vWA domain, stabilizing it in the uplifted position. (*B*) Structural modeling based on PDB 7K1J shows that once DNA-PKcs is recruited to the Ku–DNA complex, it binds directly with Ku. This interaction sterically restricts the bidirectional rotational movement of Ku along DNA, effectively “locking” Ku at the DNA end next to DNA-PKcs, as illustrated by the bolt and nut analogy. (*C*) Phosphorylation of DNA-PKcs induces conformational remodeling that permits the recruitment and activation of Artemis. Activated Artemis then gains access to DNA ends to execute its endonuclease activity. The structure shown is adapted from PDB 7SGL.

The initial event in NHEJ is the recognition of a DNA DSB by the Ku70/80 heterodimer Ku (Fig. 2A, end sensing). Ku was named after a Japanese autoimmune patient whose serum contained high levels of anti-Ku antibodies (Mimori et al. 1981). Ku70 and Ku80 are mutually dependent for their protein stability, forming an obligatory heterodimer (Gu et al. 1997b; Singleton et al. 1997; Hanakahi and West 2002). Ku-deficient mice display growth retardation, microcephaly, profound radiosensitivity, and severe combined immunodeficiency (RS-SCID) characterized by a complete absence of mature B and T lymphocytes due to failure to repair the programmed DSBs generated during V(D)J recombination (Nussenzweig et al. 1996; Zhu et al. 1996; Gu et al. 1997a). Structural analyses reveal Ku as a preformed ring that threads onto DNA ends, positioning Ku70 closer to the end and Ku80 further in (Walker et al. 2001; Chen et al. 2018). Ku fits into DNA major grooves without base-specific contacts, explaining its high affinity (*K*_*d*_ ∼10^−9^–10^−10^ M) and sequence-independent binding to double-stranded DNA (dsDNA) ends (Blier et al. 1993; Rathmell and Chu 1994a; Smider et al. 1994; Pfingsten et al. 2012) as well as its preference for B-form dsDNA over A-form dsRNA (Paillard and Strauss 1991; Blier et al. 1993; Rathmell and Chu 1994b; Smider et al. 1994; Walker et al. 2001). The motion of Ku along the dsDNA is like a bolt on a screw, rotating inward rather than sliding on (Walker et al. 2001; Chen et al. 2021b). While small stem–loops and short overhangs are all acceptable, long single-stranded overhangs or large secondary structures (e.g., stem–loops >15 bp) limit Ku loading (Rathmell and Chu 1994a; Walker et al. 2001; Dalby et al. 2013), consistent with end binding and explaining why long single-stranded DNA (ssDNA) overhangs generated by end resection during initial steps of HR are not a good substrate for Ku (Mimitou and Symington 2008; Symington and Gautier 2011). Although the core Ku70 structure required for dimer formation (von Willebrand factor A [vWA] and β barrels) is conserved from yeast to humans and vertebrates, Ku70 and Ku80 acquired additional C-terminal domains connected with the core via a flexible linker. These noncore regions, while dispensable for Ku stability, are critical for Ku mobility on DNA ends and tethering (see below). Specifically, recent structural and genetic studies have identified a role for the C-terminal SAP domain of Ku70 in DNA and nucleosome binding, potentially limiting Ku sliding and anchoring Ku in proximity to nucleosomes (Zhu et al. 2024; Eickhoff et al. 2025; Wang et al. 2025; Hall et al. 2026). Ku is essential for recruiting other NHEJ components involved in end protection, end tethering, end processing, and end ligation (Mari et al. 2006; Yano et al. 2008). Cryo-EM studies have shown that the Ku70/Ku80 von Willebrand A (vWA) domains form a wing-like structure with slight up–down mobility (Fig. 2A, right) and dedicated docking sites for PAXX and XLF C-terminal Ku binding domains (KBMs) beneath the “wing,” thereby facilitating the tethering of the two DNA ends (Grundy et al. 2016; Chen et al. 2021a, 2023). The binding of DNA-PKcs to DNA-bound Ku on the Ku70 side has also been associated with an upward conformational shift of the Ku70 vWA domain (Chen et al. 2021b), which likely facilitates the insertion of PAXX KBM-70 under the Ku70 vWA domain. Similarly, the XLF C-terminal KBM-80 inserts under the vWA domain of Ku80, which is shared among APLF, WRN, and potentially others. The handle or relatively thin cradle part of the Ku ring provides an additional docking site for LIG4, Polµ, and Polλ, as well as RAP1 in the telomere complex (Chen et al. 2021a; Eickhoff et al. 2025; Frit et al. 2025; Liu et al. 2025; Vogt et al. 2025). These Ku–protein interactions are typically low-affinity and dynamic, consistent with the flexible and modular architecture of the NHEJ complex previously noted (Ochi et al. 2014). Together, Ku functions as the ultimate sensor that detects DSBs and assembles the entire NHEJ machinery on DNA ends.

## End protection

In cells, exposed DNA ends are susceptible to nuclease and helicase attack, and end protection is critical and operates in at least two modes (Fig. 2B, end protection). On one side, Ku encircles both strands of the DNA end, preventing helicase-driven unwinding and exonuclease (e.g., EXO1)-mediated resection (Mimitou and Symington 2010; Shao et al. 2012). Genetically, loss of Ku rescues the embryonic lethality of *Lig4*^−/−^ mice by permitting end resection, thereby enabling HR and A-EJ to compensate for a subset of repair events (Karanjawala et al. 2002). Correspondingly, residual repair junctions recovered from Ku-null mice and cells often contain larger deletions (Nussenzweig et al. 1996; Zhu et al. 1996; Gu et al. 1997b; Boboila et al. 2010; Liang et al. 2021).

On the other side, DNA-bound Ku recruits the large catalytic subunit of the DNA-dependent protein kinase (DNA-PKcs), forming the DNA-PK holoenzyme (Gottlieb and Jackson 1993), which protects the ends from end ligation and terminal processing. DNA-PKcs loading also prevents Ku from sliding off in mouse cells (Zhu et al. 2024) or overloading to the ends in human cells with ∼100-fold higher Ku expression (Bossaert et al. 2024; Zhu et al. 2025). Cryo-EM structures of DNA-PKcs, Ku, and DNA show that in the absence of ATP, DNA-PKcs binding pushes Ku to rotate inward ∼10 bp (almost a full 360°). The HEAT repeats of DNA-PKcs fold into a large forceps shape, clamping the DNA ends. In addition to the shared dsDNA acting like a needle threading through both Ku and DNA-PKcs to keep them together, DNA-PKcs also interacts with the Ku70 vWA. Moreover, the C-terminal domain and the tail of Ku80 also wrap around DNA-PKcs through two interactions identified biochemically and visualized in cryo-EM structures (Fig. 2C, inset; Singleton et al. 1999; Falck et al. 2005; Chen et al. 2021b; Liu et al. 2022). Complete loss of DNA-PKcs (null) in mice is compatible with murine development and selectively blocks CJ formation due to an inability to recruit the Artemis endonuclease (see “End Processing”). In contrast, mice expressing a kinase-dead (KD) DNA-PKcs (D3922A; *DNA-PKcs*^*KD*^) die in utero with severe neuronal apoptosis, like in *Lig4*^*−/−*^ or *Xrcc4*^−/−^ mice, and are unable to repair either blunt SEs or hairpin CEs, indicative of end ligation defects (Jiang et al. 2015; Menolfi and Zha 2020). Ku deletion rescues the embryonic lethality of *DNA-PKcs*^*KD/KD*^ mice (Jiang et al. 2015), revealing an “end protection” role of DNA-PKcs that is regulated by its own kinase activity. A DNA-PKcs-deficient patient with mutations that compromise kinase activity and markedly lower DNA-PKcs expression displayed severe immune deficiency and growth retardation and succumbed to neurological complications at 36 months of age, consistent with end ligation defects beyond end processing (Woodbine et al. 2013). Biochemically, purified DNA-PKcs blocks DNA ligation by a T4 DNA ligase in the absence of hydrolyzable ATP (Block et al. 2004). Cryo-EM analyses revealed that in the ATP-free (Apo) form, the N-terminal HEAT repeats of DNA-PKcs interact with the 5′ phosphorylated terminus of the DNA end, physically preventing end ligation (Chen et al. 2021b; Zha et al. 2021), nicely explaining why *DNA-PKcs*^*KD/KD*^ mice and cells, like *Lig4*^−/−^ or *Xrcc4*^−/−^ mice, display a complete end ligation defect (Jiang et al. 2015; Crowe et al. 2018). Although DNA-PKcs can bind DNA in vitro in the absence of Ku (Ma et al. 2002; Chen et al. 2021b), particularly under low-salt conditions (50 mM vs. the physiological 120–150 mM), all known physiological functions of DNA-PKcs in NHEJ, including end tethering, end protection, and end processing, are Ku-dependent. Consistent with this, Ku knockout rescues the pathophysiological effects of phosphorylation-defective or kinase-dead DNA-PKcs (Jiang et al. 2015; Shao et al. 2020). Nevertheless, a Ku-independent function of DNA-PKcs cannot be completely excluded.

While DNA-PKcs and Ku protect DNA ends from ligation and end processing through direct binding, they cannot shield flanking DNA beyond the region physically embraced by the Ku–DNA-PKcs complex from endonucleolytic cleavage. Several pieces of evidence suggested that Ku–DNA-PKcs complexes bound to unrepaired DNA ends can serve as a barrier to activate the endonuclease activity of the MRE11–RAD50–NBS1 (MRN)–CtIP complex, which nicks the flanking DNA (Myler et al. 2017; Deshpande et al. 2020). In general, this mechanism facilitates the removal of stalled Ku and DNA-PKcs at nonligatable ends, like it does to SPO11 during meiosis (Cejka and Symington 2021), and eventually initiates end resection required for HR. A notable exception might be at the telomere, where DNA-PKcs prevents MRN-mediated resection in the presence of TRF2 and RAP1 (Myler et al. 2023; Eickhoff et al. 2025). On chromatin, endonuclease resection nearly a DNA ends is further regulated by ATM kinase and ATM-mediated DNA damage response and cell cycle. On the one hand, ATM-dependent phosphorylation of histone H2AX initiates the MDC1–RNF8–RNF168 cascade and recruits 53BP1 to protect surrounding chromatin from MRN–CtIP-mediated cleavage (Helmink et al. 2011; Zha et al. 2011a; Liu et al. 2019). On the other hand, ATM phosphorylates MRN and CtIP to promote their endonuclease activity (Sartori et al. 2007; Takeda et al. 2007; Peterson et al. 2012; Aparicio et al. 2016; Wang et al. 2021). In the S and G2 phases, CtIP level increases and CDK4/6-mediated phosphorylation of CtIP promotes endonuclease activity (Huertas et al. 2008), whereas 53BP1 preferentially binds methylated chromatin that is enriched in G0/G1 before replication (Orthwein et al. 2015; Nakamura et al. 2019), thereby balancing end protection with clearance of stalled Ku–DNA-PKcs-bound DNA ends in a cell cycle-dependent manner.

The role of DNA-PKcs in end protection is regulated by its kinase activity and its autophosphorylation. DNA-PKcs belongs to the PI3 kinase-related protein kinase (PI3KK) family, which also includes ATM, ATR kinases, and mTOR (Hartley et al. 1995; Shiloh 2003). Purified DNA-PKcs can be activated by Ku and DNA (Lees-Miller et al. 1990; Hartley et al. 1995; Ma et al. 2002) and phosphorylates itself and other substrates. Consistent with ATP hydrolysis and kinase activity being critical for relieving end protection by DNA-PKcs and allowing end ligation, DNA-PKcs-null murine cells join blunt SEs efficiently with a moderate decrease in fidelity (Bosma et al. 1988; Gao et al. 1998; Taccioli et al. 1998). DNA-PKcs is the best-understood substrate of itself, with two well-characterized phosphorylation clusters (Meek et al. 2008). The S2056 cluster is mainly autophosphorylated by DNA-PK itself (Chen et al. 2005; Meek et al. 2007; Nagasawa et al. 2011), while the T2609 cluster can be phosphorylated by ATM (Chen et al. 2007; Shrivastav et al. 2009; Yajima et al. 2009; Nagasawa et al. 2011), DNA-PK (Meek et al. 2007; Neal et al. 2016), and even the ATR kinase upon UV damage (Yajima et al. 2006). Alanine substitution of either the S2056 or T2609 phosphorylation clusters in human cells markedly impaired joining efficiency and altered junctional deletion size, indicating that DNA-PKcs phosphorylation regulates DNA end processing (Cui et al. 2005; Neal et al. 2014). However, because junctional deletion analyses in asynchronous cell populations cannot unequivocally distinguish whether the increased deletion size and reduced joining efficiency result from defects in Artemis-mediated end processing or from subsequent MRN–CtIP-initiated endonucleolytic resection, the precise role of DNA-PKcs in this transition remained unresolved until more recently. In contrast to the embryonic lethality and complete V(D)J recombination defects in *DNA-PKcs*^*KD/KD*^ mice, mice carrying homozygous alanine substitutions at either the S2056 cluster (*DNA-PKcs*^*PQR/PQR*^) (Jiang et al. 2019; Zhu et al. 2023) or the T2609 cluster (*DNA-PKcs*^*5A*^ and *DNA-PKcs*^*3A*^) (Zhang et al. 2011; Crowe et al. 2020; Shao et al. 2020) were born alive. Lymphocytes derived from *DNA-PKcs*^*5A*^, *DNA-PKcs*^*3A*^, and *DNA-PKcs*^*PQR*^ mice are largely proficient in chromosomal V(D)J recombination, with more defects associated with T2609 cluster phosphorylation deficiency (Zhang et al. 2011; Lee et al. 2013; Jiang et al. 2019; Crowe et al. 2020; Shao et al. 2020; Zhu et al. 2023). Careful analyses of V(D)J recombination junctions and general DSB lesions recovered from phosphorylation site mutant mice revealed a moderately increased deletion size at both CJs and SJs and frequency and increased use of MH at the junction, consistent with mild end ligation defects (Jiang et al. 2019; Crowe et al. 2020; Zhu et al. 2023). Loss of XLF exacerbates the V(D)J recombination and end ligation defects in mice lacking the S2056 cluster phosphorylation (*DNA-PKcs*^*PQR*^), highlighting partial functional redundancy within NHEJ (Zhu et al. 2023). The relatively mild NHEJ deficiency of individual cluster phosphorylation-deficient versus kinase-dead DNA-PKcs suggests that two phosphorylation sites might have cooperative yet distinctive roles. In this context, ATM kinase activity and ATM-mediated phosphorylation of kinase-dead DNA-PKcs enable Artemis activation and end processing in vivo but cannot displace DNA-PKcs for end ligation (Jiang et al. 2015), indicating that there are at least two different DNA-PKcs phosphorylation stages—one for end processing and one for complete release. Cryo-EM analyses show that ATP hydrolysis triggers large conformation changes in the DNA-PKcs involving the two autophosphorylation clusters, which lose the grip by N-HEAT and M-HEAT domains and reduce the DNA-PKcs–DNA interaction (Liu et al. 2022; Chen et al. 2023). Both cell biology and structural analyses support the role of DNA-PKcs autophosphorylation in releasing DNA-PKcs from the DNA ends and relieving end protection (Davis et al. 2010; Liu et al. 2022). So, why bother to recruit DNA-PKcs if it must be removed for end illation? Indeed, DNA-PKcs is not conserved in yeast and other primitive eukaryotes. The secret lies in the need for end tethering and end processing. In this context, recent structural and genetic evidence suggests that DNA-PKcs phosphorylation facilitates Artemis activation and also promotes DNA-PKcs release from DNA ends (Liu et al. 2022).

## End processing (part I: Artemis)

About 10% of radiation-generated DSBs in G1-arrested cells require end processing before ligation (Riballo et al. 2004), which could be mediated by many different mechanisms (e.g., PNK). Here, we focus on the two that strictly depend on Ku and are intricately linked to NHEJ: Artemis-mediated endonuclease cleavage (in the presence of DNA-PKcs; this section) (Fig. 2C, end processing I) and polymerase (TdT, Polµ, and Polλ)-mediated end filling (after DNA-PKcs release; see “End Processing (Part II: Polymerases)” ) (Fig. 2C, end processing II). For productive V(D)J recombination, hairpin-sealed CEs must be opened, and the overhangs must be filled in or removed (e.g., 3′ overhangs) before ligation. Hairpin opening and fill-in contribute to the formation of P and N elements, respectively, and the diversity of the antigen receptor gene loci (Lafaille et al. 1989; McCormack et al. 1989).

The hairpin opening and endonuclease processing are mediated by Artemis with the help of DNA-PKcs and its phosphorylation (Jiang et al. 2015; Liu et al. 2022). Because lymphocyte development and CJ formation require the resolution of hairpinned CEs, loss of Artemis results in T^−^B^−^ severe combined immunodeficiency (SCID) with lymphocytes arrested at the earliest step of V(D)J recombination (Moshous et al. 2001; Rooney et al. 2002). Similar phenotypes are noted for DNA-PKcs-null mice or mice with a spontaneous SCID mutation that severely compromises DNA-PKcs protein stability (Roth et al. 1992; Blunt et al. 1996; Gao et al. 1998; Taccioli et al. 1998). However, unlike Ku or *Lig4/Xrcc4*-null, *Artemis*-null or *Prkdc*-null mice are of normal size and can join blunt SEs efficiently, indicating that DNA-PKcs and Artemis are at least partially dispensable for ligation itself (Gao et al. 1998; Rooney et al. 2002). While ATP hydrolysis is required to activate Artemis in vitro, hairpin opening at CEs still occurs in *DNA-PKcs*^*KD/KD*^ cells via ATM kinase activity (Ma et al. 2002; Jiang et al. 2015). This role of ATM in Artemis activation is likely not limited to *DNA-PKcs*^*KD/KD*^ cells and might not require direct phosphorylation of Artemis itself, because ATM kinase activity also contributes to ∼10% of radiation-induced DSB repair in G1-arrested cells in a manner epistatic to Artemis (Riballo et al. 2004). Moreover, delayed CE processing likely underlies the increased hybrid joint (HJ) formation observed in ATM- and MRN-deficient cells (Bredemeyer et al. 2006; Gapud et al. 2011; Helmink et al. 2011; Zha et al. 2011b), as HJ accumulation is also seen in hypomorphic Artemis mutants (Bredemeyer et al. 2006; Huang et al. 2009). Together, these findings support a model in which ATM-mediated and DNA-PKcs-mediated phosphorylation of DNA-PKcs promotes Artemis-mediated end processing. DNA-PKcs activation and its phosphorylation (notably at the T2609 cluster) induce inward rotation of its HEAT repeat “clamps” to present the DNA hairpin terminus to Artemis (Fig. 2C, end processing I; Liu et al. 2022), which engages only ∼6 nt around the hairpin apex, while DNA-PKcs positions the nuclease for cleavage. Alanine substitution at the T2609 cluster did not completely abolish hairpin opening and V(D)J recombination unless an ATM kinase inhibitor was also included (Gapud et al. 2011; Crowe et al. 2020; Shao et al. 2020), suggesting that additional phosphorylation events also contribute. We note that the role of ATM in hairpin opening and V(D)J recombination/NHEJ is most evident in the context of chromatin DNA double-strand breaks and is not readily apparent in plasmid-based assays. One possible explanation is that ATM kinase activation, even in vitro, requires kilobases of double-stranded DNA (Paull 2015), raising the possibility that plasmid-based V(D)J recombination substrates lacking native chromatin organization fail to activate ATM efficiently. Consistent with this notion, V(D)J recombination on chromosomal substrates is substantially more efficient than that measured using plasmid-based assays.

The C terminus of Artemis that is invisible in the current structure also contains multiple PI3KK phosphorylation sites (Ma et al. 2002; Liu et al. 2022), though whether and how ATM/DNA-PKcs-mediated phosphorylation of Artemis contributes to Artemis activation remains unresolved. While highly phosphorylated in the reconstitution system, the alanine substitution at the Artemis C-terminal tail did not blunt hairpin opening (Ma et al. 2002; Goodarzi et al. 2006; Jiang et al. 2015). Thus, current data support the importance of ATM-mediated and DNA-PKcs-mediated phosphorylation of DNA-PKcs, while a contribution from phosphorylation of other substrates, including Ku and Artemis, cannot be completely ruled out. Structural studies support a primarily structural and scaffolding role for DNA-PKcs in Artemis activation. Consistent with a kinase-independent role of DNA-PKcs in Artemis activation, a patient carrying the DNA-PKcs L3062R mutation exhibits normal kinase activity but fails to open CE hairpins and develop SCID, similar to Artemis deficiency (van der Burg et al. 2009). Cryo-EM analyses showed that Artemis L372 and L375 sandwich DNA-PKcs L3062, forming a critical protein–protein interaction (Liu et al. 2022).

## End tethering

Two DNA ends must be physically brought together for ligation. In contrast to HR, where extensive base-pairing guides the template-dependent DNA synthesis and repair, NHEJ ligates two DNA ends directly, highlighting the importance of end tethering via the NHEJ complex. During V(D)J recombination, the RAG endonuclease brings the DNA together before cleavage (Kim et al. 2015; Chen et al. 2020a,b), but how NHEJ brings two distal breaks together has only recently been understood. Cell biology studies that track the mobility of endonuclease-generated DSBs in cells documented the role of Ku in keeping the DNA ends together—tethering (Soutoglou et al. 2007). Since then, five molecular bridges mediated by three proteins—DNA-PKcs (I; two bridges), XLF (II; two bridges), and PAXX (III; one bridge), all Ku-dependent—have been identified. Among them, the DNA-PKcs-mediated bridges can only occur in long-range NHEJ complexes before the dissociation of DNA-PKcs, while XLF- and PAXX-mediated bridges might function in both long- and short-range complexes for end ligation. Genetic studies highlight the redundant and critical function of end tethering. While loss of any two did not abolish NHEJ in the case of DNA-PKcs-null or XLF-null, the combined loss of any three bridges (e.g., PAXX [III] + XLF KO [IIa +I Ib] or XLF [IIa + IIb] + DNA-PKcs KO [Ia + Ib]) abolishes the end ligation in genetic models.

**Figure 3.**
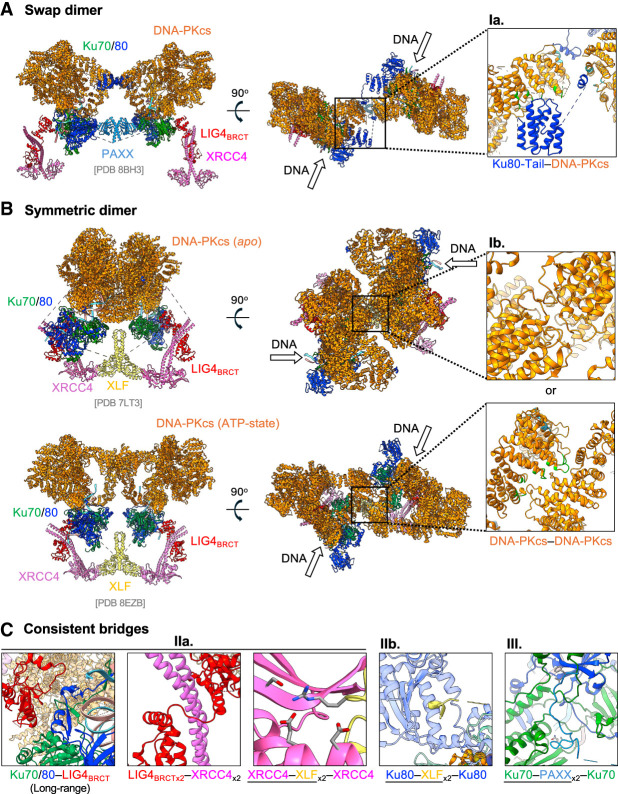
The five bridges of NHEJ. Five molecular bridges (Ia, Ib, IIa, IIb, and III) in end tethering are shown in different long-range synaptic complexes. The arrows indicate the orientation of DNA. (*A*) The cryo-EM structure of the long-range synaptic complex with PAXX shows a handshake mode (PDB 8BH3). Panel *Ia* shows the bridge that the C-terminal tail of Ku80 (blue) binds to the DNA-PKcs (orange) on the other side. (*B*) Panel *Ib* depicts the bridge association of two DNA-PKcs connecting the opposite ends. Two distinct conformations determined by DNA-PKcs phosphorylation status (*apo* and ATP state) have different DNA-PKcs–DNA-PKcs interaction modes. The Apo form of DNA-PKcs holds the DNA ends with an angular distortion (Supplemental Movie S1), which can be switched into a near-collinear orientation in the ATP state. The structural models are adapted from PDB 7LT3 and PDB 8EZB. (*C*, panels *IIa*,*IIb*,*III*) Magnified view of three bridges (IIa, IIb, and III) that stay consistent in the long-range synaptic complex. All interactions are captured from the left side of the complex in PDB 8EZB or PDB 8BH3.

The first two tethering mechanisms are mediated by DNA-PKcs and Ku and are limited to the long-range complex (Figs. 1B, 3A,B). Single-molecule analyses show that Ku and DNA-PKcs bring the two DNA ends together independent of end ligation (Graham et al. 2016). Cryo-EM analyses of Ku and DNA-PKcs in complex with DNA provide at least two conformations that support this end-tethering function. In one scenario, the C-terminal tail of Ku80, but not the C-terminal domain (helix bundle), reaches over to the DNA-PKcs on the other DNA end in a loose handshake mode known as the swap dimer/loose handshake model (Chaplin et al. 2020; Gluza et al. 2025). Notably, the same Ku80 C-terminal tail can also bind to the DNA-PKcs in *cis* at the same patch of amino acids on DNA-PKcs (at the same DNA end) and stabilize DNA-PKcs on Ku and DNA (Figs. 2C [inset], 3A; Chen et al. 2021b). In the other scenario, the two DNA-PKcses on each end can interact with each other to form a symmetric long-range synaptic complex (Chen et al. 2021a). Depending on the availability of ATP, the symmetric DNA-PKcs bridge can be in either a closed configuration that is not compatible with Artemis (Fig. 3B, top) or an open configuration potentially compatible with Artemis (Fig. 3B, bottom; Supplemental Movie S1). Notably, in the closed symmetric DNA-PKcs mode, the interaction surface between the two DNA-PKcses was where the C-terminal domain of Ku80 bonded to (Figs. 2C [inset], 3B [top]), while in the open symmetric DNA-PK mode, the interaction surface is the same as that bound by Ku80 tails in the loose handshake mode or in *cis* (Figs. 2C [inset], 3A,B [bottom]). In the swap dimer/loose handshake mode, the two Kus and DNA-PKcses are further away and rotate relative to each other (Chaplin et al. 2020). While both scenarios can be found in the single-molecule cryo-EM preparation, the two scenarios cannot occur simultaneously. The interchange between the two tethering modes might reflect a dynamic sampling at the initial phase of NHEJ. This Artemis-independent and kinase activity-independent end-tethering function of DNA-PKcs protein might explain the reduced SJ fidelity in DNA-PKcs-null (98% reduced to ∼85%) but not Artemis-null murine lymphocytes (Gao et al. 1998; Rooney et al. 2002).

**Figure 4.**
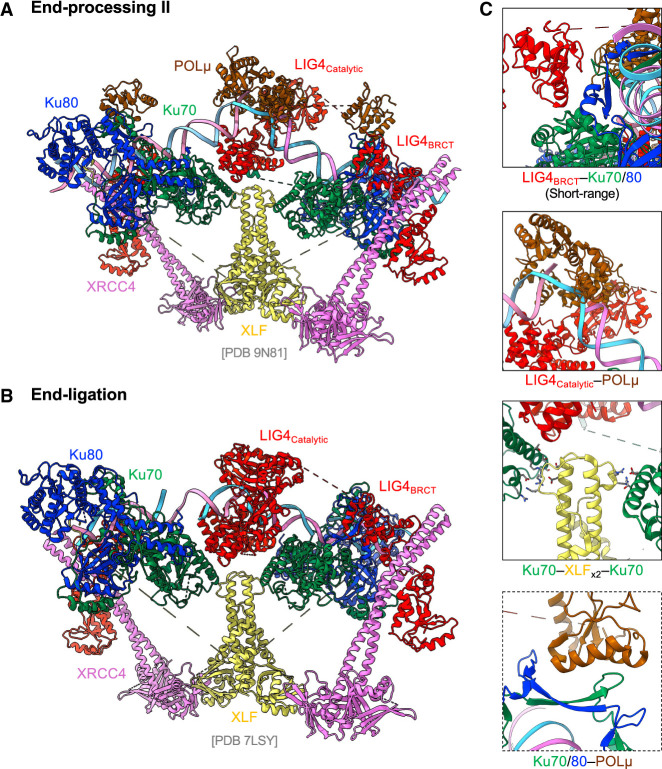
The molecular model for end processing II and end ligation. (*A*) The end processing II model for gap filling is adapted from the cryo-EM structure PDB 9N81. Polµ is shown as a representative polymerase, where Polλ and TdT can also be similarly involved. (*B*) The cryo-EM structure PDB 7LSY illustrates the core functional complex required for end ligation. In the absence of DNA-PKcs, the single catalytic domain of LIG4 (LIG4_catalytic_) is visualized with an unligatable DNA nick. (*C*) Magnified view of additional bridges observed in the short-range synaptic complex. All interactions are captured from the complex in PDB 9N81.

The third and fourth end-tethering mechanisms are mediated by XLF, which forms two independent bridges between DNA ends (Figs. 1B, 3B,C). XLF's interaction with XRCC4 depends on DNA (Lu et al. 2007), and the yeast ortholog of XLF (Nej1) also plays a role in end ligation (Valencia et al. 2001), suggesting that XLF might be part of the end ligation complex. However, in contrast to the more severe and inborn RS-SCID caused by defects in other previously characterized NHEJ genes (e.g., *Artemis*, *LIG4*, or *PRKDC*) (Moshous et al. 2001; van der Burg et al. 2006, 2009), XLF deficiency in patients causes autosomal recessive immunodeficiency with variable severity and relatively delayed onset (often at nearly 10 years old) (Dai et al. 2003; Ahnesorg et al. 2006; Buck et al. 2006). Moreover, two mouse models of XLF deficiency were born alive, in contrast to the embryonic lethality of Lig4 or Xrcc4 deficiency in mice, and display only moderate defects in V(D)J recombination (Li et al. 2008a; Vera et al. 2013). Finally, NHEJ measured by the repair of I-SceI-induced reports also recapitulated the “moderate” role of XLF in end ligation in comparison with *LIG4*-null (Gunn and Stark 2012; Carney et al. 2020). This phenotypic difference is explained by the realization that XLF contributes to end ligation during NHEJ through end tethering (Fig. 3B,C). Structurally, XLF forms a bean sprout-shaped homodimer, reminiscent of XRCC4, and binds XRCC4 dimers via their head domains (Ahnesorg et al. 2006; Li et al. 2008b; Mahaney et al. 2013). Unlike XRCC4, however, XLF carries a flexible C-terminal tail containing a Ku80-binding motif (KBM-80)—a feature shared with APLF. The XLF tail–Ku80 vWA domain interaction might stabilize the Ku80 vWA domain in the uplifted/open configuration (Figs. 2A, 3C, panel IIb). Notably, the Ku80-binding motif (less than five amino acids) is significantly shorter than the Ku70-binding motif (KBM-70) found in PAXX (Grundy et al. 2016) and the two are not interchangeable (Fig. 3C, panel IIb vs. panel III). Single-molecule studies first suggested, and cryo-EM later visualized, that one XLF dimer bridges the two DNA ends (Graham et al. 2016, 2018; Carney et al. 2020) through two routes: First, the C-terminal tail of XLF inserts under the Ku80 vWA domain, directly linking two Ku80s (Fig. 3C, panel IIb; Graham et al. 2018; Carney et al. 2020; Chen et al. 2021a); second, the XLF head domain bridges two XRCC4 dimers, which bind to LIG4 via their respective stems (Fig. 3B,C, panel IIa). Each LIG4 then interacts with Ku at each end to form a large ω-shaped framework crucial for the final ligation step (Fig. 3B; Graham et al. 2018; Carney et al. 2020; Chen et al. 2021a). Finally, we note that in the short-range complex, after DNA-PKcs displacement, the end of the XLF stem could potentially engage with Ku directly to provide an additional end-tethering role (Fig. 4).

Despite XLF's critical role in end tethering, its function is surprisingly partially redundant with the long-range Ku–DNA-PKcs bridge and the PAXX complex, as only the combined loss of XLF and DNA-PKcs or PAXX abolishes signal joint formation during V(D)J recombination (Oksenych et al. 2013; Kumar et al. 2016; Lescale et al. 2016; Tadi et al. 2016; Hung et al. 2017; Liu et al. 2017). In addition to redundancy with the core NHEJ mechanism discussed here, the vulnerable NHEJ in XLF-deficient cells helped reveal a critical role of chromatin-associated DNA damage in NHEJ. XLF-deficient cells are substantially impaired for V(D)J recombination of plasmid-based substrates, but XLF-deficient lymphocytes develop normally and join chromosomal RAG-initiated DSBs during V(D)J recombination at nearly wild-type levels (Li et al. 2008a), suggesting functional redundancy with chromatin-associated DNA damage response. Accordingly, it was later discovered that ATM kinase and its chromatin-bound substrates in DNA damage response (53BP1, H2AX, and MDC1) all play an essential role in supporting chromosomal NHEJ and V(D)J recombination in XLF-deficient cells (Liu et al. 2012; Oksenych et al. 2012, 2013; Beck et al. 2020). Given the end-tethering role of XLF in core NHEJ (Fig. 3B,C), it is tempting to speculate that ATM and its substrates might also promote NHEJ in XLF-deficient cells by providing a stable and favorable chromatin environment. Moreover, ATM kinase phosphorylates the MRE11–RAD50–NBS1–CtIP complex to promote end resection, which competes with 53BP1 and H2AX and promotes homology-dependent repair at the price of NHEJ (Helmink et al. 2011; Zha et al. 2011a; Liu et al. 2012; Oksenych et al. 2012).

The fifth known tethering mechanism (Figs. 1B, 3A,C, panel III) is mediated by the paralog of XRCC4 and XLF (PAXX). Two groups independently identified PAXX while looking for new NHEJ proteins via pull-down assay using XRCC4 as a bait (Ochi et al. 2015; Xing et al. 2015). As its name implies, PAXX also forms a bean sprout-shaped homodimer. The C-terminal tail of each PAXX contains an ∼10 amino acid Ku70-binding motif (KBM-70), which inserts under the vWA domain of Ku70 at each end (Chen et al. 2023; Seif-El-Dahan et al. 2023; Gluza et al. 2025). In contrast to the Ku80 vWA shared by XLF, APLF, and WRN, PAXX is the only known protein that has KBM-70 thus far. Unlike XLF, PAXX is not known to interact with XRCC4 and only contributes one bridge, explaining the relatively moderate role of PAXX in NHEJ compared with XLF. Correspondingly, loss of PAXX alone does not affect chromosomal V(D)J recombination or cause primary immunodeficiency in mouse models (Lescale et al. 2016; Tadi et al. 2016; Hung et al. 2017; Liu et al. 2017). PAXX has not been linked to human primary immunodeficiency either. Unlike the Ku80(vWA)–XLF dimer–Ku80(vWA) bridge, the Ku70(vWA)–PAXX dimer–Ku70(vWA) bridge anchors on Ku70 near the ends and is compatible with the swap dimer/handshake model of Ku–DNA-PKcs interaction, suggesting that PAXX might be involved in both long-range complexes (Chen et al. 2023; Seif-El-Dahan et al. 2023). In this context, DNA-PKcs binding to the Ku70 side of the Ku ring lifts the Ku70 vWA, likely promoting PAXX–Ku70 interaction. In addition, PAXX tail insertion might stabilize the Ku70 vWA in the uplifted configuration (Fig. 2A). The partial redundancy and overlapping between XLF and PAXX in end tethering explains why the loss of PAXX or XLF alone has only a moderate impact on chromosomal V(D)J recombination, while the loss of both completely abrogates end joining, V(D)J recombination, and murine embryonic development (Kumar et al. 2016; Lescale et al. 2016; Hung et al. 2017; Liu et al. 2017). Moreover, loss of PAXX is also synergistic with DNA-PKcs loss (Xing and Oksenych 2019), supporting a role of PAXX in the long-range complex shortly after DNA-PKcs binding and before XLF engagement.

Notably, all three tethering mechanisms and five bridges require Ku as the sole anchor on the DNA ends (Figs. 1B, 3), explaining dramatic increases in end diffusion in Ku-deficient cells observed in early cell biology experiments (Soutoglou et al. 2007). While the DNA-PKcs–Ku-mediated tethering must dissolve before end ligation (see “End Protection”), the tethering by XLF and PAXX is maintained in the short-range complex and is compatible with end ligation. Consistent with the order, dissociation of DNA-PKcs often precedes the complete resolution of XLF/LIG4 in single-molecule (Reid et al. 2015; Graham et al. 2016; Zhao et al. 2019) and cell biology (Uematsu et al. 2007; Davis et al. 2010; Mikhova et al. 2024) analyses. The five bridges mediated by DNA-PKcs, XLF, and PAXX function cooperatively and partially redundantly to ensure efficient end ligation, highlighting the importance of end tethering for NHEJ, in which the two DNA ends share no or minimal complementation. While here we described five well-characterized bridges, additional tethering mechanism might exist. Based on their synergy with XLF, ERCC6L2 (Francica et al. 2020; Liu et al. 2020), CYREN/MRI (Arnoult et al. 2017; Hung et al. 2018), and H2AX/53BP1 (Zha et al. 2011a; Liu et al. 2012; Oksenych et al. 2012) might also contribute to the end tethering directly or indirectly. Loss of tethering would not only compromise productive V(D)J recombination but also increase IR sensitivity and chromosomal translocation, as the broken DNA ends would diffuse into other chromosomal territories and participate in error-prone repair. Accordingly, in a Tp53-deficient background, mouse models lacking NHEJ factors, including Ku and DNA-PKcs, develop immature B-cell lymphomas with IgH-Myc translocations (Zhu et al. 2002; Rooney et al. 2004).

## End processing (part II: polymerases)

Artemis cleaves the CE hairpin at either the apex or 2–4 nt 5′ or 3′ of the apex (Meier and Lewis 1993; Ma et al. 2002; Liu et al. 2022), which might require filling or resection before ligation. Similarly, radiation- and endonuclease-generated breaks might not be blunt and require processing. While Polµ and LIG4 can bind to the Ku cradle even in the presence of DNA-PKcs, polymerase-mediated fill-in can only occur after DNA-PKcs displacement, which allows polymerases (Polµ and Polλ) and LIG4 to access the DNA in the short-range ligation complex. LIG4–Ku binding is essential for the ω frame as well as the tethering bridge formed by Ku–LIG4–XRCC4–XLF–XLCC4–LIG4–Ku (Fig. 4A, end processing II). Moreover, the XLF stem now is in the range to directly interact with Ku70 (Fig. 4C, third panel), and the PAXX bridge remains. Presumably, after DNA-PKcs displacement, LIG4 can access Ku70 in addition to the cradle. LIG4 binds to the DNA breaks and, together with its interaction with Polµ, stabilizes both LIG4 and Polµ on Ku and effectively prevents DNA-PKcs rebinding, thereby securing the transition to the short-range complex (Fig. 4C). Mammalian Ku is known to directly recruit DNA Polμ and Polλ for template-dependent end filling at 5′ overhangs at the CEs. Recently, structural studies have suggested that one Polμ and one Polλ can interact with Ku and LIG4 on opposite site ends at the same time; as such, they could take turns to complete the end processing (Liu et al. 2025; Vogt et al. 2025). Deletion of Polμ or Polλ does not abrogate V(D)J recombination or lymphocyte development, but the loss of both reduces the variable region junction length (Bertocci et al. 2006). In addition, human and mouse lymphocytes also express a development stage-specific X family polymerase, terminal deoxynucleotidyl transferase (TdT), during V(D)J recombination (Alt and Baltimore 1982). TdT is not required for V(D)J recombination, as lymphocyte counts are normal in Tdt-deficient mice (Gilfillan et al. 1993; Komori et al. 1993). Biochemically, TdT adds nontemplated nucleotides to free 3′ hydroxyls like DNA polymerase. Thus, end processing, especially the processing of the hairpin-sealed coding ends by the collective action of DNA-PKcs, Artemis, Polμ, Polλ, and TdT both before and after DNA-PKcs displacement, markedly enhances the diversity of V(D)J recombination junctions and Ig/TCR repertoires. Notably, this high level of “imprecision” is mostly dictated by the hairpin ends and TdT and is not inherent to the ligation mechanism of the NHEJ pathway. The SJs formed by two blunt SEs are almost always precise (>90% fidelity in vivo and on plasmid substrates) (Li et al. 2008a; Wang et al. 2020).

## End ligation

After DNA-PKcs dissociation from the end and polymerase fill-in are complete (see above), the ends are ready for ligation, which is accomplished through the activity of the XRCC4/LIG4 complex with the help of XLF and PAXX for tethering (Fig. 4A,B, end ligation). Although there are three known mammalian ligases, LIG1, LIG3, and LIG4, all of which can ligate double-stranded DNA ends in vitro (Ellenberger and Tomkinson 2008), neither LIG1 nor LIG3 can compensate for LIG4 during NHEJ and the repair of V(D)J recombination-generated DSBs (Frank et al. 1998; Grawunder et al. 1998a). This is partly due to the partner and the unique recruitment mechanism of LIG4. In cells, LIG4 forms a complex with its obligatory partner, XRCC4 (Grawunder et al. 1998b). Specifically, two XRCC4 molecules form a bean sprout-shaped homodimer, and LIG4 binds to the stem of the XRCC4 dimer via its BRCT domains. During NHEJ, one XRCC4 dimer binds onto each side of an XLF dimer via their respective head domains (Ropars et al. 2011; Mahaney et al. 2013; Roy et al. 2015), contributing to another layer of end tethering through the LIG4–Ku interaction on each end. If Polµ or Polλ is available, LIG4 interaction with the polymerase could also further stabilize each other (Liu et al. 2025; Vogt et al. 2025). In contrast, at the leading strand telomere, RAP1, recruited to the ends by sequence-specific telomere binding protein TRF2, competes with LIG4 for Ku binding, explaining why Ku and DNA-PKcs fail to ligate telomere ends in normal conditions (Eickhoff et al. 2025). LIG4, like LIG1 and LIG3, must be readenylated before each round of ligation (Riballo et al. 2009). Moreover, the repair of a DNA double-strand break necessitates two LIG4 molecules to accommodate the antiparallel polarity of the DNA strands given their specific interaction with Ku (Chen et al. 2021a). The presence of one XRCC4 dimer at each side of XLF allows each XRCC4 dimer to bring one LIG4 to the break to complete the ligation of both strands. Neither LIG1 nor LIG3 has a known sequential/pair-loading mechanism. This difference might explain why LIG4 is uniquely suited for DSB repair and NHEJ. XRCC4 is required for the protein stability of LIG4 (Bryans et al. 1999; Jiang et al. 2015). However, unlike the interdependence of the protein stability seen in the Ku70/Ku80 heterodimer, XRCC4 protein is stable in the absence of LIG4 (Cottarel et al. 2013; Francis et al. 2014), though in this context, XRCC4 appears to be almost entirely sequestered to the cytoplasm, thus making any direct function in end joining unlikely (Francis et al. 2014). Mice deficient for either Xrcc4 or Lig4 are essentially indistinguishable. Deficiency for Xrcc4 or Lig4 leads to late embryonic lethality associated with apoptosis of newly postmitotic neurons in the developing nervous system (Frank et al. 2000; Gao et al. 2000). Loss of the *Tp53* tumor suppressor gene successfully rescues the embryonic lethality (Frank et al. 2000; Gao et al. 2000), suggesting that the lethality is likely the result of a Tp53-dependent response to unrepaired DSBs. However, while organismal viability is restored, lymphocyte development is not; thus, similar to Ku-deficient mice, Tp53-deficient and Xrcc4-deficient or Lig4-deficient mice exhibit an RS-SCID phenotype, where developing lymphocytes are arrested at the progenitor stage due to the inability to repair RAG-initiated DSBs during V(D)J recombination. Given these observations, it is likely that XRCC4/LIG4 functions in V(D)J recombination and NHEJ as part of a requisite complex. XLF, the latest NHEJ member cloned from primary immunodeficiency patients, was originally considered the third member of the ligation complex (Dai et al. 2003; Ahnesorg et al. 2006; Buck et al. 2006). However, as discussed above in the context of end tethering, XLF interacts with the XRCC4–LIG4 complex in a DNA-dependent manner, and its primary role in end ligation is to promote and stabilize end tethering through the formation of the ω-shaped framework.

## Summary

The NHEJ pathway, though fully reconstituted on naked DNA substrates (Ramsden et al. 1997; Gellert et al. 1999), operates physiologically in chromatin, where DNA double-strand breaks (DSBs) activate the ATM-dependent DNA damage response (DDR). ATM phosphorylates and promotes the recruitment of chromatin substrates such as H2AX, 53BP1, and shieldin to stabilize DNA ends and ensure accurate repair (Shiloh 2003, 2014; Callén et al. 2009; Ghezraoui et al. 2018; Noordermeer et al. 2018). Although dispensable in plasmid assays, ATM shows strong genetic synergy with XLF and DNA-PKcs, underscoring its central role in chromosomal end joining (Callén et al. 2009; Gapud et al. 2011; Zha et al. 2011a,b). ATM's kinase activity also directly activates Artemis, explaining the V(D)J recombination-associated repair defect in *Atm*-null mice that is absent in H2AX- or shieldin-deficient models (Borghesani et al. 2000; Celeste et al. 2002, 2003; Franco et al. 2006; Callén et al. 2007; Difilippantonio et al. 2008; Zha et al. 2010; Hu et al. 2014). In addition, ATM coordinates two opposing processes: end protection and end resection. Through phosphorylation of H2AX and 53BP1, ATM establishes the shieldin complex to protect ends and favor NHEJ over homologous recombination (Bunting et al. 2010; Polato et al. 2014; Xu et al. 2015; Gupta et al. 2018; Noordermeer et al. 2018). Conversely, ATM-mediated phosphorylation of CtIP promotes the MRN-dependent endonuclease resection required for homology-directed repair when breaks persist into S/G2 (Yu and Baer 2000; Huertas et al. 2008; Helmink et al. 2011; Peterson et al. 2011; Zha et al. 2011a; Liu et al. 2012, 2019; Oksenych et al. 2012). Thus, ATM fulfills at least four roles in NHEJ: activating Artemis, stabilizing and tethering ends via 53BP1/H2AX, protecting ends through shieldin, and initiating resection via MRN–CtIP when NHEJ repair fails.

Recent advances in cryo-EM and genetics have allowed us to reconstruct a step-by-step process of NHEJ at each end—from end sensing by Ku, to end protection by Ku and DNA-PKcs, to end processing through DNA-PKcs phosphorylation and Artemis recruitment and activation. Through a less-understood process involving DNA-PKcs's kinase activity and phosphorylation, DNA-PKcs dissociates from the ends, which allows NHEJ-associated DNA polymerases and LIG4 to load onto the junctions (Fig. 1A). Throughout the NHEJ process, five dynamic bridges by DNA-PKcs, XLF, and PAXX keep the two ends together through end tethering (Fig. 1B). Overall, the success of NHEJ relies on a network of weak, dynamic, yet redundant interactions, particularly during end tethering, which distinguishes this pathway from homology-based repair. Emerging players such as PAXX, ERCC6L2, and CYREN/MRI (Arnoult et al. 2017; Hung et al. 2018; Francica et al. 2020; Liu et al. 2020) further underscore the pathway's extended regulatory network and function in mammalian cells as genome expansion and postmitotic life expectancy increase. Outstanding questions include how DNA-PKcs dissociates from the Ku-bound DNA, how Ku and the NHEJ complex are disassembled after ligation, how the Artemis C-terminal region and TdT regulate end processing, how the many different proteins with KBMs (e.g., WRN and APLF) contribute to NHEJ, and how NHEJ is selectively regulated and suppressed at telomeres via TRF2-mediated recruitment of RAP1 and Apollo (van Overbeek and de Lange 2006; Sonmez et al. 2024; Eickhoff et al. 2025). The evolutionary acquisition of Ku70/80 C-terminal domains may also reflect an adaptation to larger genomes and longer cellular life spans. Future single-molecule and structural studies will be key to resolving the dynamic assembly and disassembly of the NHEJ machinery.

## Supplemental Material

Supplement 1
